# Bibliometric Analysis on the Risks of Oral Cancer for People Living with HIV/AIDS

**Published:** 2017-11

**Authors:** Luana Kelle Batista MOURA, Mitra MOBIN, Francisca Tereza Coelho MATOS, Thiago Lima MONTE, Eliana Campelo LAGO, Carlos Alberto Monteiro FALCÃO, Maria Ângela de Arêa Leão FERRAZ, Tanit Clementino SANTOS, Fabrício Ibiapina TAPETY, Carla Maria de Carvalho Leite Leal NUNES, Laelson Rochelle Milanês SOUSA

**Affiliations:** 1. Dept. of Family Health, University Center UNINOVAFAPI, Teresina, Piauí, Brazil; 2. Universidade Estadual do Maranhão, UEMA, Teresina, Piauí, Brazil; 3. Universidade Estadual do Piauí, UESPI, Teresina, Piauí, Brazil; 4. Dept. of Nursing, Federal University of Piauí, Piauí, Brazil

## Dear Editor-in-Chief

In recent years, viral infections as risk factors for the onset of oral cancers have been well discussed by the international academic-scientific community. In Brazil, according to the National Cancer Institute, in 2016, 15490 new cases of oral cancer were estimated, counted as 11140 men and 4350 women (1).

Bibliometry as a data collection and analysis technique has been used as an argumentative source in the search for investment resources in researches (2–3). The ISI Web of Knowledge, Web of Science^TM^ database was used in this study for its international academic recognition to be considered one of the most comprehensive journals covering several areas of scientific knowledge (4). Data collection from the ISI Web of Knowledge/Web of Science^TM^ database was performed using descriptors: oral cancer, HIV and Aids and exported to the HistCite^TM^ bibliometric analysis software to organize information and facilitate analysis.

According to Table 1, the search resulted in 201 articles on oral cancer and HIV/AIDS in the English, German and French languages, published in 111 different journals indexed to the main database and written by 954 authors with links to 340 institutions located in 37 countries. For the achievement of these articles, 7021 references were used, with an average of approximately 35 references per article (Table 1).

**Table 1:** General Results of the Bibliometric Survey on oral cancer and HIV/AIDS (1989–2015). Teresina – PI, 2016

***General bibliometric data***	***Records***
Records (papers)	201
Indexed journals	111
Authors	954
Institutions (authors links)	340
Countries	37
Languages	3
Cited references	7.021

Source: Elaboration based on data from the Web of Science.

**Fig. 1:**
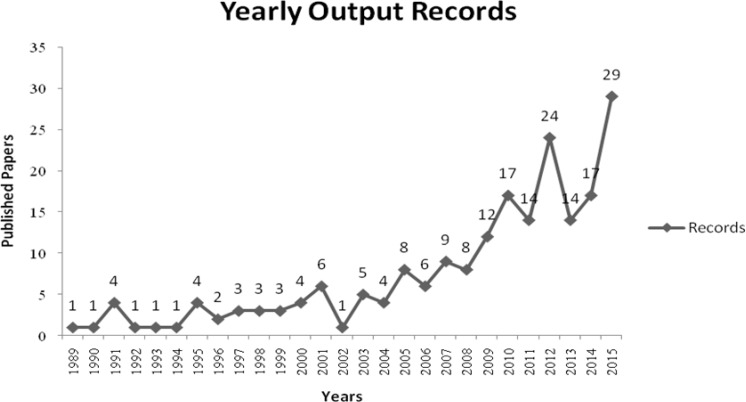
Frequency distribution of publications on oral cancer and HIV/AIDS (1989–2015). Teresina – PI, 2016 Source: Elaboration based on data from the Web of Science.

As it is showed in the amount of publication per year (Fig. 1), the first published paper dates from the year 1989. Until 1995, the publication record is relatively low, reaching a maximum of 4 records. There is a considerable increase in the number of records starting in 2012, in relation to previous years, with 24 published papers. The year of 2015 stands out with 29 records..

The journals with the highest number of records are presented in Table 2. The “Oral Oncology” and “Journal of the American Dental Association” with 16 and 10 papers, respectively, are highlighted. The most prominent journal in relation to the number of publications is specialized in publications related to head and neck neoplasias. The second journal is specialized in publications focused on dentistry. Although it is the second in number of publications, it is the first one with greater impact, presenting a citation index by amount of publications of 48, 8.

**Table 2:** Journals with more records (1989–2015). Teresina – PI, 2016.

***Journals***	***Number of papers***	***Citations***	***Citations/Number of papers***
Oral Oncology	16	684	42,7
Journal of The American Dental Association	10	488	48,8
Head and Neck-Journal for The Sciences and Specialties of The Head and Neck	7	218	31,14
International Journal Of Oral And Maxillofacial Surgery	7	182	26,0
Journal of Oral Pathology & Medicine	7	38	5,4
British Journal of Oral & Maxillofacial Surgery	6	66	11
British Dental Journal	4	73	18,25
Clinical Oral Investigations	4	8	2
European Journal of Cancer Prevention	4	74	18,5
Oral Diseases	4	73	18,25
Oral Surgery Oral Medicine Oral Pathology Oral Radiology And Endodontics	4	163	40,75

**Source:** Elaboration based on data from the Web of Science.

The paper distribution by the countries of origin of the authors, with more records is presented in Table 3. Despite the majority characteristic of the United States, it is possible to observe a distribution of scientific production to other America’s countries, such as Peru, in addition to other continents: Europe, Australia and Asia.

**Table 3:** Number of papers by country of origin of the authors (1989–2015). Teresina – PI, 2016

***Country***	***Records***
USA	67
UK	36
India	20
Germany	15
Japan	11
Canada	10
Taiwan	9
Australia	7
Brazil	7
France	7
Italy	7

**Source:** Elaboration based on data from the Web of Science.

The 16 articles with the greatest impact were published in the period 1995–2010. Of these, the study published in 2008 (5) in the journal Oral Oncology, is the most cited by the authors of the 201 articles in the sample. Among the 201 papers, the study published in 2008 (6) in the journal European Journal of Surgical Oncology was the most cited in the Web of Science Database during the period.

Thus, this study have shown the evolution of publications in 26 yr of building scientific knowledge about oral cancer risks of people living with HIV/AIDS and can contribute to the development of new researches to fill knowledge gaps and implement effective public policies all around the world.
